# Exploring Novel Non-pharmacologic Approaches to Address Preoperative Anxiety and Postoperative Pain in Pediatric Patients Undergoing In-Patient Surgical Procedures: A Scoping Review

**DOI:** 10.7759/cureus.52006

**Published:** 2024-01-10

**Authors:** Gabriela E Llerena, Emily Krzykwa, Michael Huzior, Nicole Vilar, Danielle Donahue, Hanan Zisling, Patricia Zielinski, Nisarg Shah, Tara Lewandowski, Stanley Dennison, Noel Alonso

**Affiliations:** 1 Medicine, Nova Southeastern University Dr. Kiran C. Patel College of Osteopathic Medicine, Fort Lauderdale, USA; 2 Pediatrics, Nova Southeastern University Dr. Kiran C. Patel College of Osteopathic Medicine, Fort Lauderdale, USA

**Keywords:** anxiety, children, non-pharmacological, pediatric, pediatric surgery, postoperative pain, scoping review

## Abstract

The pediatric population is more prone to experiencing anxiety and fear before undergoing an inpatient surgical procedure than adults. Non-pharmaceutical interventions, such as music therapy and virtual reality programs, have shown significant promise in reducing the post-operative pain associated with pre-operative anxiety of patients and their caregivers. While there is evidence to support the use of non-pharmaceutical treatment in the mitigation of pre-operative anxiety, there are limited published reports of non-pharmacological interventions for pre-operative anxiety in children undergoing inpatient surgical procedures. The goal of this scoping review was to identify and classify specific non-pharmacological interventions utilized inpatient among children to improve pre-operative anxiety and post-operative complications inflicting pain. Comprehensive searches were conducted using Ovid Medline, Embase Emtree, CINAHL Complete, and COCHRANE Central databases. The articles had to be peer-reviewed, written in English, published between 2000-2022, and contain measurements of pre-operative anxiety and post-operative pain to be included in the scoping review. Articles that reported findings on patients younger than 18 undergoing elective and/or routine surgeries, excluding emergent surgical cases, were selected. After a systemized screening process, 9 articles were selected for the final review. The findings indicated that non-pharmacological interventions such as virtual reality, hypnosis, and clowns reduced pre-operative anxiety and post-operative pain in pediatric patients. This scoping review identified a wide range of non-pharmacological interventions to mitigate the post-operative effects of pre-operative anxiety among children, including but not limited to music, visual reality, and other holistic methods. More longitudinal studies are warranted to understand the specific interventions that may be the most efficacious.

## Introduction and background

Millions of surgical procedures are performed annually in the United States, and many patients may experience some uneasiness before their operation. Surgical interventions, especially for those who lack a clear understanding of operative procedures, are stressful and anxiety-provoking. In certain types of surgery, anxiety may even increase post-operative morbidity and mortality [1]. Several factors may influence feelings of pre-operative anxiety, including fears of anesthesia, pain, or complications. Pediatric populations especially are prone to these fears and often experience a considerable amount of anxiety leading up to surgery. Each year in the United States (U.S.), 4.7% of children aged 0-17 undergo surgical procedures, accounting for 3.9 million procedures performed annually [2]. 

Children are universally regarded as a vulnerable and impressionable population. Events during childhood that provoke fear or perceived danger can have long-term effects on children, such as increased fear and perceived danger around medical settings. Despite knowledge of these long-term impacts, there has been little research surrounding pre-operative anxiety in children [1]. Anxiety is an adaptive response to a threat and can manifest in physiological and psychological disturbances. Anxiety can be assessed clinically through blood pressure, heart rate, and cortisol measurements [2]. The use of questionnaires can also be helpful as a non-physical measurement of anxiety [2]. Seventy-five percent of children undergoing surgical procedures experience pre-operative anxiety, and 65% of children experience negative behavioral changes post-operatively [2]. Early recognition and management of anxiety can help improve surgical outcomes and avoid potential complications, including increased post-operative pain and sleep disturbances [1]. Pre-operative emotional support coupled with familiarization with surgical techniques and hospitalization was shown to reduce anxiety [3]. Increasing awareness of the complications related to pre-operative anxiety has led to new techniques to ease patient's nerves, specifically non-pharmacologic therapies. Increasing awareness of the complications related to pre-operative anxiety has led to new techniques to ease patient's nerves, specifically non-pharmacologic (e.g., music therapy, meditation, and play therapy)

Visualization and meditation interventions

Premedication is the most common intervention to reduce pre-operative anxiety, with midazolam used most frequently [4]. Midazolam belongs to the benzodiazepine class of medications, which are central nervous system depressants and anxiolytics that are used in inpatient and outpatient settings. Midazolam is traditionally used perioperatively as anesthesia induction, maintenance, and sedation. Midazolam is the preferred benzodiazepine for anesthetic use due to its rapid, nonpainful induction and lack of irritation compared to other benzodiazepines. In a study with 126 children aged between 5-12 years old, anxiety levels were measured across three randomly assigned groups: painting therapy, premedication with midazolam, and a control group. The children in the painting group were guided to draw what they imagined about the operating room for 30 minutes, while the children in the premedication group received IV midazolam for 2-3 minutes before being transferred to the operating room [4]. The control group did not receive any interventions before the operation. The study revealed that anxiety levels were lowest in the painting group, demonstrating that painting therapy could serve as a viable, cost-effective alternative to premedication to reduce pre-operative anxiety in children [4]. A similar study was performed in which a group of preschool-aged children were given animated surgery-related picture books one week before surgery versus a control group. The study revealed that preschool-aged children had decreased pre-operative anxiety when given an animated picture book explaining their upcoming surgery compared to those who did not receive an animated picture book [5]. Lastly, meditation effects on anxiety were examined in adolescents. Both studies investigated the use of meditation, specifically mindfulness meditation (MM) and transcendental meditation (TM), and demonstrated decreased anxiety levels in experimental groups utilizing MM and TM meditation compared to the control groups who did not use any meditative practices [6]. 

Musical interventions

Musical interventions, such as music therapy and music medicine, are two cognitive therapies that have been gaining popularity due to the negative side effects that anti-anxiety medications can have on patients [7,8]. Music medicine is categorized as passively listening to pre-recorded music, whereas music therapy requires implementing a music-therapeutic process and using personally tailored music experiences [7,9,10]. Several studies have shown that music therapy can significantly reduce several physiological as well as psychological factors that lead to pre-operative anxiety in patients of all populations. However, overall, the results have been inconclusive [11]. Specifically, in the study performed by Kain et al., reduction in pre-operative anxiety via music therapy did not demonstrate a strong correlation with the music itself but rather with the music therapists aiding the study. This was demonstrated by the two music therapists in the study who provided the same intervention, but the results revealed reduced pre-operative anxiety with one of the therapists but not with the other. Therefore, alluding to the theory that the therapists themselves may have a greater impact on the therapy than initially thought.

Additionally, studies specifically focusing on pediatric patients aged 10-19 years of age have demonstrated that although music therapy can create a relaxing and calming environment, solely using it to reduce pre-operative anxiety does not have significant effects in comparison to anti-anxiety drugs or sedatives. However, there are confounding variables found within many pediatric studies, and the results should be taken cautiously. Although musical therapy research has been inconclusive thus far, attention is being paid to several other cognitive therapies.

Game/play interventions

In addition to pre-operative musical interventions, there have been developments in more interactive modules to reduce pre-operative anxiety in the pediatric population. Exposure to virtual reality (VR) programs before operations has been shown to reduce anxiety and improve patient and physician satisfaction [12,13]. In one study that utilized VR programs, the children randomized to the VR group were allowed to choose between viewing a program in which they were walking through the Amazon forest or a program that simulated waterskiing [12]. Measurements of pre-operative versus post-operative anxiety exhibited statistically significant lower levels of anxiety in children who utilized the VR programs as distractions before the procedure [12]. Additionally, administering these virtual programs as a part of the patient care process has been shown to reduce the need for analgesics in the post-operative setting [14]. These findings have significant implications as they provide evidence of a novel, non-pharmacologic intervention for reducing stressors in the clinical environment [15]. It is well documented that depression and anxiety are associated with worsened surgical outcomes; therefore, the deployment of novel modalities to reduce depression and anxiety is warranted [16]. 

Gaps in the literature

The current literature on reducing pre-operative anxiety in pediatric populations is limited in that the concept of mental and physical health being integrated is relatively new to medicine. It has been documented, however, that early recognition and intervention of pre-operative anxiety in patients and their caregivers is effective in reducing potential complications, making pre-operative anxiety a concern of physicians for these reasons. The use of novel therapeutic relaxation techniques, such as musical interventions and interactive virtual reality programs, shows significant promise in reducing the morbidities associated with pre-operative anxiety of both the patients and their caregivers. There are an increasing number of studies being published regarding new non-pharmacological ways to reduce anxiety and pain in patients, but there is minimal literature discussing their feasibility, cost, or efficacy in comparison amongst each other. Additionally, this study revealed that the caregiver's degree of concern before surgery was also found to be a strong influence on the occurrence of pre-operative anxiety [17], a factor that is not widely described in current literature. This scoping review aimed to identify and conclude the efficacy of non-pharmacological strategies to reduce pre-operative anxiety in pediatric populations and, in turn, minimize post-operative pain.

## Review

Methods 

Eligibility Criteria

The inclusion criteria for this review were based on whether the study provided information on complementary and/or alternative medicine or therapies, included pediatric populations, and discussed pre-operative anxiety on post-operative pain. The articles had to be peer-reviewed, written in English, and published between 2000-2022 to be included in the scoping review. Both qualitative and quantitative studies were used. Articles that collected data on patients younger than 18 years of age who experienced pre-operative anxiety prior to surgery were included, as well as patients undergoing elective and/or routine surgeries (excluding emergency surgical cases). Articles that assessed pharmacological and/or medical treatments [only] on pre-operative anxiety were excluded. 

Search Procedures

The research team established the search criteria for the study. Once the search parameters pertaining to the research question were established, a set of key terms related to the topic was generated. This included different spellings of the keywords, finding synonyms for the words, and allowing for different grammatical tenses of the words to be searched for. Once this was established, the team searched and gathered relevant articles from four online databases. All searches were conducted in September 2022. 

Search Strategy

The review question was established based upon a Population, Concept, and Context (PCC strategy), with the population being 28 children under the age of 18, the concept being those being treated for their anxiety before surgery with a non-pharmacological method, and the context being the overall change (if any) of their post-operative pain. Based on this strategy, the research question developed is as follows: "What novel, non-pharmacological interventions have been successful in eliminating or decreasing the level of pre-operative anxiety in children to reduce the levels of post-operative pain?" The databases Ovid-Medline, Embase-Emtree, CINAHL Complete, and COCHRANE-central databases were chosen due to their reliability, comprehensiveness, and ability to cover different types of journals. The aim was to collect as many relevant articles as possible to establish a comprehensive basis to begin the reviews. 

Once the databases were selected, team members conducted the searches using standardized descriptors for each database, which pertained to our research question. These descriptors were expanded upon through the use of different spellings of each individual word, synonyms, Boolean operator AND and OR, as well as identifying individual attributes that pertained to one phrase, which would have otherwise caused an article to be excluded if it had not been modified.

Search Terms

The search phrase for CINAHL Complete was "anxiety AND children OR adolescents OR adolescent OR youth OR child OR teenager OR pediatric OR pediatric OR kids OR infants AND pre-operative OR pre-operative OR pre-op AND post operative OR post-operative OR post-operative OR post surgery OR post-surgical OR post-surgical AND pain OR discomfort OR distress AND non-pharmacological interventions OR non-pharmacological therapy OR non- pharmacological treatment OR Virtual reality." These search terms were then used in the databases Ovid Medline, Embase Emtree, and COCHRANE Central per the database parameters.

Screening and Study Selection

**Figure 1:**
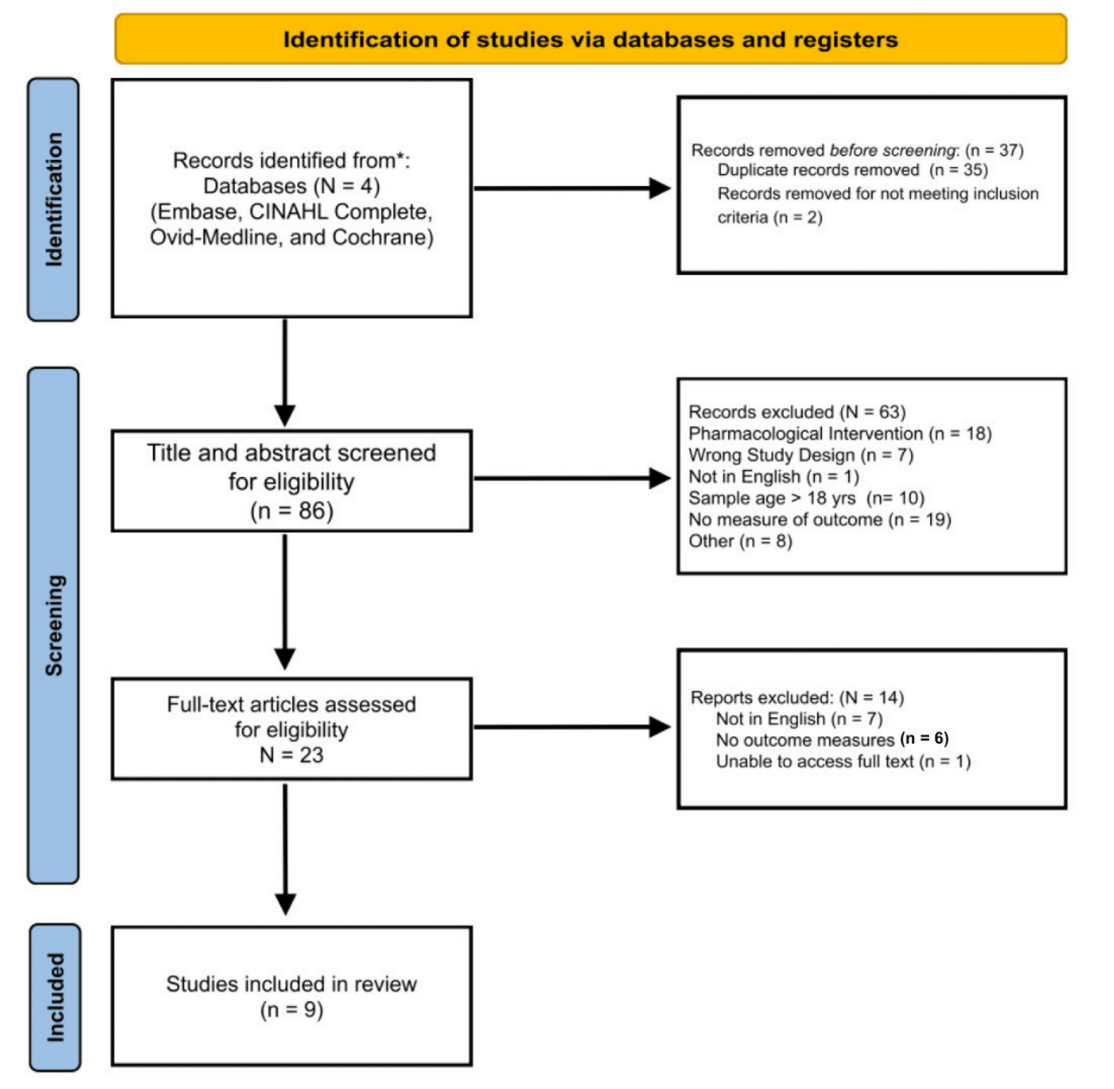
PRISMA flow diagram PRISMA: Preferred Reporting Items for Systematic Reviews and Meta-Analyses

The initial search yielded 123 citations. Once duplicates (n = 35) and other articles not meeting inclusion criteria (n = 2) were removed, 86 unique citations were left to be screened for eligibility. The team conducted a first-line screening process whereby the titles and abstract were reviewed for relevance. From this process, 23 articles remained for further evaluation by reading the full text. Discrepancies were resolved by another team member who served as an independent reviewer to break any decision ties. This process resulted in the exclusion of 14 articles, resulting in nine for the final review. Articles that were not in English (n = 7), had no outcome measure (n = 6), and were inaccessible (n = 1) were excluded. The screening and selection process is depicted in Figure 1. 

Data extraction

In order to assess the effectiveness of the non-pharmacological interventions to lower pre-operative anxiety and post-operative pain, quantification methods for pre-operative anxiety and post-operative pain were required. Data were extracted from articles on general study information (publication date, study location, and publication language), characteristics of the sample (age and sex), procedures (types of medical inpatient procedures), quantifications (measurements of pre-operative anxiety and post-operative pain), and interventions (types of non-pharmacological interventions).

Results

Characteristics of Included Studies

The articles were grouped based on the non-pharmacological mechanism utilized in the study as well as based on the effects noticed, specifically whether or not the mechanism altered pre-operative anxiety and/or post-operative pain. All articles were written in English and conducted in the following countries: United States (n = 2), Turkey (n = 2), France (n = 1), Italy (n = 1), Israel (n = 1), the Netherlands (n = 1), and China (n = 1). Four followed a randomized study design, and seven were performed as randomized controlled trials. The studies included various age ranges, with seven studies examining children under the age of 12 years, one study examining children from ages 2 -16, and three studies examining children 10 - 18 years old. The oldest article included was conducted in 2003, and the most recent article included was conducted in 2021.

Once the articles were identified, the type of intervention used and anxiety/pain scales were categorized into one of three categories: Cognitive Interventions (n = 5), Visual Interventions (n = 4), and Auditory Interventions (n = 1). The pain scales used in the articles included the Wong-Barker Faces Scale (n = 2), the Face, Legs, Activity, Cry, Consolability (FLACC) Scale (n = 3), the Faces Pain Scale-Revised/Numerical Rating Scale (FPS-R/NRS) (n = 1), the Visual Analogue Scale (VAS) (n = 2), and Self-reported/observed (n = 1). The anxiety scales used included the Children's Fear Scale (n = 1), the Confusion Assessment Method-Severity (CAM-S) (n = 1), the Modified Yale Pre-operative Anxiety Scale (m-YPAS) (n = 5), the Visual Analogue Scale (VAS) (n = 2), the Chinese version of the State Anxiety Scale for Children/Children's Emotional Manifestations Scale (CSAS-C/CEMS) (n = 1), the State-Trait Anxiety Inventory (STAI) (n = 1), the Spielberger's (1983) State-Trait Anxiety Inventory (n = 1), and self-reported Numerical Rating Scale (n = 1).

Cognitive Interventions

Articles were categorized based on the interventional modalities utilized. Five articles utilized cognitive interventions (i.e., relaxation-guided imagery, clown exposure, hypnosis, therapeutic play, concrete objective information, and coping information). Relaxation-guided imagery, which is a focused relaxation that helps create harmony between the mind and body in study conducted by Vagnoli where in a randomized study with 60 participants (age range 6 - 12 years [18]. The m-YPAS and FLACC Scale were used to measure pre-operative anxiety and post-operative pain, respectively. Participating children were randomly assigned to the experimental group or control group. Results from this study demonstrated that the pre-operative anxiety and post-operative pain levels were significantly lower in the experimental group compared to the control group. 

The incorporation of medical clowns was facilitated by the study conducted in Kocherov through a randomized controlled trial (N=80), with the ages ranging from 2 to 16 years old [19]. The m-YPAS and FLACC Scale were utilized to measure pre-operative anxiety and post-operative pain, respectively, in children who were undergoing meatotomies. Children were randomly assigned to the experimental group, where they interacted with clowns who, along with the parents, accompanied them throughout the process, or the control group, where only the parents accompanied the children throughout the procedure. Results from this trial showed that clown exposure yielded a lower pre-operative anxiety score, required less induction time for the anesthesia, required less time to recover from the surgery, spent overall less time in the operating room, and were discharged early. Pain scores favored the experimental group, but the result was not statistically significant when compared to the control group. When asked about their preference for medical clowns, 96.43% of health professionals favored having the clowns in the operating theater, and 97.5% of parents favored continuing to use medical clowns as an intervention.

Hypnosis was another modality studied to determine its impact on post-operative anxiety and post-operative pain. Duparc-Alegria conducted a randomized clinical study (N=120; age range 10-18 years) [20]. The VAS and FPS-R/NRS were used to measure pre-operative anxiety and post-operative pain, respectively. Children scheduled for major orthopedic surgery were randomly assigned to a hypnosis group or a control group, which received a short hypnosis session. Results from this study showed that there was no difference between the control group and the hypnosis group when it came to post-operative pain and anxiety, but there was a significant decrease in anxiety between the day before the surgery and the day after surgery. 

A randomized study by Li and colleagues used therapeutic play as an intervention for children aged 7-12 undergoing day surgery [21]. Of the sample size 203, 97 children were randomly assigned to the experimental group. Children in the experimental group were provided one hour of therapeutic play one week prior to surgery, which involved observing a demonstration of a doll receiving oxygen and anesthesia. Then, they were allowed to touch and play with the medical equipment. Results from this study showed a statistically significant lower anxiety score in children in the experimental group and exhibited less of an emotional response at the induction of anesthesia compared to the control group. There was no statistically significant difference in post-operative pain scores between the experimental and control groups. The Children's Emotional Manifestation Scale (CEMS) was utilized to measure anxiety, and the VAS was used to measure pain. 

Cognitive-behavioral interventions, which utilize guided imagery, relaxation, and positive thinking, were also studied as a means of reducing pre-operative anxiety and post-operative pain. LaMontagne conducted a randomized study that utilized four groups: control, concrete-objective information-only intervention, coping-only instruction intervention, and combined information plus coping intervention [22]. The groups that received coping intervention were provided with videos about relaxation, positive thinking, and deep breathing exercises that would help with pain and anxiety relief. The groups that received concrete-objective information were provided with videos on specific details about the surgical procedure, anesthesia, and the bodily sensations the children could experience related to the surgery. Anxiety was measured using the STAI and CSAS scales, and pain was measured using the VAS.

The control and two single modality intervention groups were combined into a single redefined group and compared to the information plus coping strategy group. The information plus a coping group showed a statistically significant reduction in pre-operative anxiety compared to the redefined group. There were significant reductions in post-operative pain in the control, information-only, and information-plus coping groups. When controlling for age, the interventions that included coping strategies were more effective in reducing post-operative pain in adolescents 13 years old and younger. 

Of the five subtypes of cognitive interventions used, all had an influence on pre-operative anxiety and/or post-operative pain in some regard. Studies using relaxation and cognitive-behavioral therapy interventions showed statistically significant reductions in both parameters, whereas studies using clown exposure, hypnosis, and therapeutic play showed a reduction in pre-operative anxiety with no statistically significant reduction in pain. 

Visual Interventions

Along with cognitive interventions, visual interventions such as virtual reality exposure and educational animated movies were examined as non-pharmacological interventions. However, unlike cognitive interventions, the results were not as homogenous as two of the five studies identified did not demonstrate a significant decrease in pre-operative anxiety and post-operative pain. 

The educational animated movie intervention was examined by Binay Yaz in a randomized controlled trial with a sample size of 132, with the ages ranging from 6-12 years old [15]. The Wong-Baker Faces Pain Scale, and the Children's Fear Scale were utilized to measure pre-operative anxiety and post-operative pain, respectively, in children who were undergoing minor surgeries. The children were distributed using block randomization into three groups: Educational Animation Group (n = 44), Documentary Group (n = 44), and Control Group (n = 44). Results from this trial indicated that of the three groups, educational animated movies significantly decreased pre-operative anxiety and post-operative pain. Along with these results, the trial concluded that along with pre and post-operative anxiety and pain, respectively, educational animated movies demonstrated an increase in the child's education and cooperation with the surgery. 

The incorporation of virtual reality exposure (VRE) was examined by Buyuk and Eijlers [14, 23]. The article by Buyuk and colleagues reported on a randomized controlled experimental study (N = 78) with children aged 5-10 [23]. This study utilized the Wong-Baker Faces pain scale and the Children's Anxiety Meter State (CAM-S) scale to measure pre-operative anxiety and post-operative pain, respectively, in children undergoing surgical circumcision. In comparison, the article by Eijlers and colleagues (N =191) reported a randomized control trial with children 4-12 years old [14]. The modified Yale Pre-operative Anxiety Scale (mYAPS) and a self-reported and observed pain scale were utilized to measure pre-operative anxiety and post-operative pain in children undergoing elective day surgery, while Buyuk and colleagues used VRE as a distraction tactic during the circumcision surgery [23]. Eijlers and colleagues also used VRE as a child-friendly immersive experience to allow the patients to become accustomed to the environment and anesthetic procedures [14]. The results from these studies indicate that VRE as a distraction technique may lead to significantly lower scores during the pre and post-operative periods, whereas VRE as an immersive experience demonstrated no significant difference during the anesthetic period or self-reported pain score post-operatively.

Auditory Interventions

The final method reported in this review by Nelson and colleagues involved music-assisted relaxation techniques to help reduce pain and anxiety in children undergoing a spinal fusion for adolescent idiopathic scoliosis [24]. This article reported results from a study designed to address the immense pain that children faced post-operatively and taught them coping techniques to utilize after the surgery. The researchers developed and presented the patients with a 12-minute video that included 1) a description of music-assisted therapy in the pediatric hospital, 2) a description of music-assisted relaxation, 3) a demonstration and opportunity to practice music-assisted relaxation techniques, and 4) a sample session with a model spinal fusion patient. The study occurred in a large midwestern (in the U.S.) tertiary hospital and included 44 participants between the ages of 10-19 years randomly assigned to the control or treatment group. Data collection included self-reported pain and anxiety before and after the music therapy session and the observation of pain behaviors (indicating relaxation or distress to assess pain intensity). Individual pain and anxiety change scores (the difference between self-reporting before therapy started and immediately after therapy) were the primary outcome measures. A 0-10 numeric rating scale (NRS) was used to measure the variables of pain and anxiety; the researchers also used the Faces, Legs, Activity, Crying, and Consolability Pain Scale (FLACC) while also tracking the number of PCA (patient-controlled analgesia) attempts as another indicator of pain intensity [24]. 

Both the treatment and control groups had significant decreases in pain and anxiety following music therapy. Despite the treatment group having slightly greater differences in pain and anxiety, the changes were not statistically significant. While attempting to track the number of PCA attempts to measure pain intensity, technical difficulties lead to too much missing data. The observations of pain documented by the researchers were made every 30 seconds, with the most frequently observed behaviors being: calm, relaxed, and fidgeting. The frequency of these behaviors among the control and treatment groups was similar and not statistically significant. Table 1 reports the characteristics of the 9 articles included in the review [24].

**Table 1:** Summary table of the 9 articles in the review RCT = Randomized Controlled Trial; FLACC = Face, Legs, Activity, Cry, Consolability; FPS-R/NRS = Faces Pain Scale-Revised/Numerical Rating Scale; VAS = Visual Analogue Scale; CAM-S = Confusion Assessment Method-Severity; m-YPAS = Modified Yale Preoperative Anxiety Scale; CSAS-C/CEMS = Chinese version of the State Anxiety Scale for Children/ Children’s Emotional Manifestations Scale; STAI = State-Trait Anxiety Inventory.

References	Country of Origin	Study Design, Participants	Sample (age in years)	Type of Intervention used	Pain/Anxiety Scales	Study Objective	Study Findings	Study Limitations	Recommendations for future studies
Eijlers, R. et al. 2019 [14].	The Netherlands	RCT n = 191	4-12	virtual reality exposure (VRE)	Anxiety - mYPAS Pain - self reported and observed	This study investigated how virtual reality exposure (VRE) as a preoperative tool could affect the anxiety, pain, and delirium levels of children undergoing elective day surgery compared with a control group receiving care as usual (CAU)	Virtual Reality Exposure was found to not have a beneficial effect on anxiety, pain, and emergence delirium, however, demonstrated that children that underwent this therapy required significantly fewer analgesics post-painful surgeries. Given the associated side-effects of analgesics, this finding was clinically important.	Does not include female children. Does not include children under the age of 4 or over the age of 12.	Expanding the study to include children from 12-18 years as well. Distinguishing the results of females and males to see if gender has any effect on the effectiveness of the intervention
Vagnoli, L. et al. 2019 [18].	Italy	Randomized Study n = 60	6-12	Relaxation-guided imagery (combination of behavioral and cognitive interventions)	Anxiety - mYPAS Pain - FLACC	This study aimed to investigate the effectiveness of relaxation-guided imagery, in reducing both preoperative anxiety and postoperative pain in children undergoing minor surgery.	Results demonstrated that relaxation-guided imagery may be considered a tool to support the reduction of preoperative anxiety and postoperative pain in children who have previously had poor surgical experiences. It can be used alone without the need for any other non-pharmacological intervention that would entail additional time and costs.	Small sample size. Does not include children under the age of 6 and over the age of 12.	Expanding the study to include children from 2-6 and 12-18 years as well. Distinguishing the results of females and males to see if gender has any effect on the effectiveness of the intervention
Kocherov, S. et al. 2016 [19].	Israel	RCT n =80	2-16	Clown exposure	Anxiety - mYPAS Pain - FLACC	This study investigated the potential benefits of the participation of medical clowns in an outpatient pediatric penile surgery program.	The patients that experienced exposure to the clowns, demonstrated a lower preoperative anxiety index upon and after surgery, required less induction time for anesthesia, spent overall less time in the operating room, and required less time to recover from the surgery and to be discharged. Therefore, it was concluded that clown exposure to children preoperatively is an excellent non-traditional anxiolytic that can be utilized instead of medicated options.	Small sample size. Does not include female children. Does not include children over the age of 16.	Preforming a follow up study to examine if the same effect that clowns had on males before penile surgery (1) extends to all types of surgical procedures and (2) will have similar effect on female children
Duparc-Alegria, N. et al. 2018 [20].	France	Randomized Study n = 120	10-18	Hypnosis	Anxiety - VAS Pain - FPS-R/NRS	This study investigated how short hypnotic sessions could impact postoperative anxiety and pain in major orthopedic surgery.	Each group experienced a significant decrease in anxiety levels between the day before surgery (Day-1) and the day after surgery. This finding, however, is thought to be due to nurse pre-operative interviews and optimization in communication within the operating room. Despite these findings, it was concluded that short hypnosis sessions performed prior to a major surgery showed no difference in postoperative anxiety and pain levels.	Does not include children under the age of 10.	Expanding the study to include children from 2-10 years as well to see if it had the same effect on younger children. Distinguishing the results of females and males to see if gender has any effect on the effectiveness of the intervention
Li et al. 2007 [21].	China	RCT n = 203 Gender = females (63) and males (140)	7-12	Therapeutic play	Anxiety - CSAS-C/CEMS Pain - VAS	This study examined the effects of therapeutic play prior to surgery on postoperative pain and anxiety levels in children. The treatment group was provided therapeutic play prior to surgery while the control group received routine pre-surgical information	Children in the experimental group of a therapeutic play intervention had significantly lower state anxiety scores in pre- and postoperative periods, and exhibited fewer negative emotions during anesthesia induction, than children in the control group. No significant differences were found between the two groups in postoperative pain.	Far less females enrolled in the study. Does not include children under the age of 7 or over the age of 12.	Assessing the intervention in children undergoing major and/or more invasive surgery/ Distinguishing the results of females and males to see if gender has any effect on the effectiveness of the intervention
LaMontagne, LL. et al. 2003 [22].	United States	RCT n = 89 Gender = females (88) and males (1)	11-18	concrete-objective information, coping information, and a combination of concrete-objective information and coping information	Anxiety - Spielberger’s (1983) State-Trait Anxiety Inventory Pain - VAS	The study investigates the effectiveness of providing cognitive-behavioral information and coping instructions in different capacities on postoperative pain and anxiety in adolescents undergoing major orthopedic surgery.	Cognitive-behavioral interventions designed to prepare adolescents for surgery should be tailored to individual factors and developmental needs, especially the adolescents’ preoperative anxiety level and age.	Small sample size. Far less males enrolled in the study. Does not include children under the age of 11	Expanding the sample size, including more males, presentations by older adolescents who have gone through coping with spinal surgery. Distinguishing the results of females and males to see if gender has any effect on the effectiveness of the intervention
Binay Yaz (2021) [15].	Turkey	RCT N =132	6-12	Educational animated movies	Pain - Wong-Baker Faces Anxiety - Children’s Fear Scale	This study aimed to investigate the effects of watching an educational animated movie in the preoperative period and how it would affect children's preoperative level of fear and postoperative level of pain.	Educational animated movies were effective in reducing preoperative fear and postoperative pain and increased the educational effectiveness and cooperation of the child.	Does not include children under the age of 6 and over the age of 12.	Expanding the study to include children from 2-6 and 12-18 years as well to see if it yields similar results. Distinguishing the results of females and males to see if gender has any effect on the effectiveness of the intervention
Buyuk, E.T. et al. 2021 [23].	Turkey	Randomized Controlled Experimental Study n = 78	5-10	VR Intervention	Pain - Wong-Baker Faces Anxiety - CAM-S	This study aimed to examine the effects of using virtual reality (VR) intervention before circumcision on the pre-and postoperative anxiety and fear levels and postoperative pain symptoms in children.	Using a Virtual Reality Intervention as a distraction method before circumcision decreased children's anxiety and fear both before and after the surgery. Pain symptoms were also found to be lower during the postoperative period.	Small sample size. Does not include female children. Does not include children under the age of 5 or over the age of 10.	Expanding the study to include children from 10-18 years as well; expanding the study to analyze effects in female children
Nelson, K et al. 2016 [24].	United States	Randomized Study n = 44	10-19	Music-assisted relaxation with controlled breathing and imagery	Anxiety - self reported NRS Pain - FLACC	The study investigated the effectiveness of using music-assisted relaxation to lessen the postoperative pain and anxiety during a child’s first out of bed experience following spinal fusion surgery.	When comparing pre and post-intervention, music therapy demonstrated a large effect on both groups in terms of pain and anxiety scores. The results indicate that music therapy treatment is crucial in lowering pain intensity, regardless of pre-training.	Small sample size. Does not include children under the age of 10. Only tested on one kind of procedure.	Expanding the sample size and age ranges of the participants of the study to include those under the age of 10. Seeing if the same results can be reproduced with other surgical procedures.

Discussion

The 9 articles in this review describe the implications of non-pharmacological interventions on post-operative anxiety and pain. Comparing the studies demonstrated that those who utilized a cognitive approach or had a cognitive component to their interventions had a greater likelihood of decreasing post-operative pain and anxiety in pediatric patients.

Cognitive Interventions

In addition to influencing post-operative pain and anxiety, the studies that utilized cognitive interventions also altered the patient's attitudes regarding their upcoming procedure and their overall hospital experience. It was found that pediatric patients who had previously endured a negative surgical experience benefitted from relaxation-guided imagery as a tool to calm their anxiety [18,22]. Along with the relaxation-guided imagery, exposure to a clown and the use of therapeutic play prior to surgery was favored by parents and healthcare providers, as it reduced the anxiety experienced by these patients [21,23]. Additionally, the study that utilized clown exposure was found to decrease post-surgical hospitalization length [19]. In contrast, the study that applied hypnosis contained a confounding variable of nurse interaction with the patient, which may have skewed the positive outcome, as it was not determined whether the outcome of reduced post-operative anxiety and pain was due to the hypnosis, nurse optimism, or a combination of the two [20].

Visual Interventions

Most of the visual interventions reviewed failed to show a statistically significant difference between the study groups. However, there was a notable finding in the study performed by Buyuk and Eijlerset through virtual reality exposure (VRE) before the procedure to educate the patient on what the procedure entailed and the environment they would be entering [14,23]. A less interactive visual intervention, educational animated movies, produced the same results [23]. While focusing on a visual component, these two interventions also incorporated a cognitive approach by providing education to the patient. The education portion of these interventions proved vital to their success by allowing patients to incorporate the unfamiliar stimuli of a surgical procedure in their heads into something that made sense and was less daunting.

Auditory Interventions

The studies that used auditory interventions found no statistically significant differences between the groups and thus had less efficacy than cognitive and visual interventions. One of the studies by Nelson presented an obstacle of technical difficulties, which could have significantly affected the study's outcome [24]. As only one study was found to use auditory interventions as their main modality, further studies should be conducted to measure the true effectiveness of this type of intervention.

Limitations to Studies in the Review

Amongst the three interventional methods used, there were two prominent limitations found. The first is the small sample size amongst various studies, and the second is the focus on a singular gender within the sample population. Across many of the studies in this scoping review, it was recognized that both of these limitations were present simultaneously.

In addition to these limitations, it is worth mentioning that the pain and anxiety measurement scales, the surgical techniques utilized, and the invasiveness of the procedures lacked standardization. This is evident because no singular scale was used in more than five studies, and the interventions were applied to various surgical techniques with varying levels of invasiveness. As there tends to be a correlation between the degree of invasiveness and post-operative pain, the lack of standardization among the studies could potentially impact the overall results. On account of this, one could only generalize the results of a study to the specific procedure it pertained to. Another limitation would be the attitudes of healthcare professionals involved in the patient's care team towards non-pharmacologic interventions and accessibility to resources, which can affect both the patient's and provider's receptiveness to interventions.

Limitations to Review Process

Possible limitations of the review process could arise from the involvement of the reviewers in the study themselves. Every research team member took part in the initial article selection phase, which introduced the potential that other valuable research studies may not have been selected. Additionally, the articles were published in different countries where cultural and healthcare practices may vary, interfering with the ability to generalize the results to the U.S. (or any other) healthcare system. Lastly, given that the topic of this review is regarding "emerging" non-pharmacological interventions, there may be new studies in the near future that offer conflicting results.

Implications for Future Practice

The recognized limitations can be addressed through further studies by standardizing pain/anxiety scales, increasing sample sizes, diversifying gender enrollment, and comparing methods used across different surgical operations with the same level of invasiveness. Doing this would allow for a direct comparison of cognitive, visual, and auditory interventions and for the true impact of these non-pharmacologic interventions to be explored. We recommend that these future studies focus on more cognitive-based interventions, particularly, as the results have shown to be more successful in reducing post-operative anxiety and pain out of the three techniques.

Even with the noted limitations, the implementation of non-pharmacologic interventions may provide a clinical benefit to the post-operative anxiety and pain experienced by pediatric patients. These interventions could be used in addition to traditional pharmacologic treatments and may result in a reduced need for medication and possibly quicker recovery times. The findings can also be expanded to further evaluate their impacts on adult patients undergoing surgical procedures.

Implications for Further Research

Future research should focus on the efficacy of non-pharmacological techniques concerning cost and feasibility in practice. Although one intervention may be more efficacious than another, a cost-benefit analysis is frequently not reported in these studies. Due to this, it is difficult to make a recommendation for a non-pharmacological intervention in reducing pre-operative anxiety and post-operative pain in comparison to the standard treatment of care. Future research would also benefit from longitudinal studies where patients of different races, sexes, and ages who underwent non-pharmacologic interventions are evaluated during post-operative follow-ups for anxiety and pain levels. This would provide a greater sense of the longevity of the effects of non-pharmacological interventions.

## Conclusions

Given the vast amount of pediatric surgeries performed annually in the United States, exploring non-pharmacological interventions to lower pre-operative anxiety is encouraged, given that lower anxiety levels have a direct correlation to shorter hospitalization times and faster recoveries. Auditory and visual interventions did not show statistically significant improvements in pre-operative anxiety. Visual interventions also showed no statistically significant improvement, except through the use of virtual reality exposure, which lowered anxiety in both the pre and post-operative periods. Of the cognitive interventions included in this study, the use of relaxation techniques and cognitive behavioral therapy yielded the greatest reduction in pre-operative anxiety and post-operative pain, whereas hypnosis, medical clown exposure, and therapeutic play resulted in reductions only in pre-operative anxiety. Virtual reality programs are the most promising novel non-pharmacologic intervention to reduce pre-operative anxiety in children aged 4-12 years. Further research on the implementation of auditory and visual interventions is needed to determine efficacy on post-surgical pain and anxiety.

## References

[1] Kain ZN, Mayes LC, Caldwell-Andrews AA, Karas DE, McClain BC (2006). Preoperative anxiety, postoperative pain, and behavioral recovery in young children undergoing surgery. Pediatrics.

[2] Rabbitts JA, Groenewald CB (2020). Epidemiology of pediatric surgery in the United States. Paediatr Anaesth.

[3] Fassler D (1980). Reducing preoperative anxiety in children: information versus emotional support. Patient Couns Heal Edu.

[4] Babaei S, Fatahi Babani S, Fakhri M (2021). Painting therapy versus anxiolytic premedication to reduce preoperative anxiety levels in children undergoing tonsillectomy: a randomized controlled trial. Indian J Pediatr.

[5] Yang Y, Zhang M, Sun Y, Peng Z, Zheng X, Zheng J (2022). Effects of advance exposure to an animated surgery-related picture book on preoperative anxiety and anesthesia induction in preschool children: a randomized controlled trial. BMC Pediatr.

[6] Blum H, Rutt C, Nash C, Joyce V, Buonopane R (2021). Mindfulness meditation and anxiety in adolescents on an inpatient psychiatric unit. J Health Care Chaplain.

[7] Bradt J, Dileo C, Shim M (2013). Music interventions for preoperative anxiety. Cochrane Database Syst Rev.

[8] Wang SM, Kulkarni L, Dolev J (2002). Music and preoperative anxiety: a randomized, controlled study. Anesth Analg.

[9] Pittman S, Kridli S (2011). Music intervention and preoperative anxiety: an integrative review. Int Nurs Rev.

[10] Cooke M, Chaboyer W, Schluter P, Hiratos M (2005). The effect of music on preoperative anxiety in day surgery. J Adv Nurs.

[11] Kain ZN, Caldwell-Andrews AA, Krivutza DM, Weinberg ME, Gaal D, Wang SM, Mayes LC (2004). Interactive music therapy as a treatment for preoperative anxiety in children: a randomized controlled trial. Anesth Analg.

[12] Gold JI, Annick ET, Lane AS, Ho K, Marty RT, Espinoza JC (2021). “Doc McStuffins: Doctor for a Day” virtual reality (Docvr) for pediatric preoperative anxiety and satisfaction: pediatric medical technology feasibility study. J Med Internet Res.

[13] Jung MJ, Libaw JS, Ma K, Whitlock EL, Feiner JR, Sinskey JL (2021). Pediatric distraction on induction of anesthesia with virtual reality and perioperative anxiolysis: a randomized controlled trial. Anesth Analg.

[14] Eijlers R, Dierckx B, Staals LM (2019). Virtual reality exposure before elective day care surgery to reduce anxiety and pain in children: a randomised controlled trial. Eur J Anaesthesiol.

[15] Binay Yaz Ş, Bal Yilmaz H (2022). The effects of designing an educational animation movie in virtual reality on preoperative fear and postoperative pain in pediatric patients: a randomized controlled trial. J Perianesth Nurs.

[16] Pompe RS, Krüger A, Preisser F (2020). The impact of anxiety and depression on surgical and functional outcomes in patients who underwent radical prostatectomy. Eur Urol Focus.

[17] Liang Y, Huang W, Hu X, Jiang M, Liu T, Yue H, Li X (2021). Preoperative anxiety in children aged 2-7 years old: a cross-sectional analysis of the associated risk factors. Transl Pediatr.

[18] Vagnoli L, Bettini A, Amore E, De Masi S, Messeri A (2019). Relaxation-guided imagery reduces perioperative anxiety and pain in children: a randomized study. Eur J Pediatr.

[19] Kocherov S, Hen Y, Jaworowski S, Ostrovsky I, Eidelman AI, Gozal Y, Chertin B (2016). Medical clowns reduce pre-operative anxiety, post-operative pain and medical costs in children undergoing outpatient penile surgery: a randomised controlled trial. J Paediatr Child Health.

[20] Duparc-Alegria N, Tiberghien K, Abdoul H, Dahmani S, Alberti C, Thiollier AF (2018). Assessment of a short hypnosis in a paediatric operating room in reducing postoperative pain and anxiety: a randomised study. J Clin Nurs.

[21] Li Li, HCW HCW, Lopez V, Lee TL (2007). Effects of preoperative therapeutic play on outcomes of school-age children undergoing day surgery. Res Nurs Health.

[22] LaMontagne LL, Hepworth JT, Cohen F, Salisbury MH (2003). Cognitive-behavioral intervention effects on adolescents' anxiety and pain following spinal fusion surgery. Nurs Res.

[23] Buyuk ET, Odabasoglu E, Uzsen H, Koyun M (2021). The effect of virtual reality on Children's anxiety, fear, and pain levels before circumcision. J Pediatr Urol.

[24] Nelson K, Adamek M, Kleiber C (2017). Relaxation training and postoperative music therapy for adolescents undergoing spinal fusion surgery. Pain Manag Nurs.

