# Correction: Exploring Novel Non-pharmacologic Approaches to Address Preoperative Anxiety and Postoperative Pain in Pediatric Patients Undergoing In-Patient Surgical Procedures: A Scoping Review

**DOI:** 10.7759/cureus.c330

**Published:** 2025-10-01

**Authors:** Gabriela E Llerena, Emily Krzykwa, Michael Huzior, Nicole Vilar, Danielle Donahue, Hanan Zisling, Patricia Zielinski, Nisarg Shah, Tara Lewandowski, Stanley Dennison, Noel Alonso

**Affiliations:** 1 Medicine, Nova Southeastern University Dr. Kiran C. Patel College of Osteopathic Medicine, Fort Lauderdale, USA; 2 Pediatrics, Nova Southeastern University Dr. Kiran C. Patel College of Osteopathic Medicine, Fort Lauderdale, USA

This article has been corrected to fix the following typo in the PRISMA diagram: 'Reports excluded' and 'Studies included' corrected from 12 to 14 and 11 to 9, respectively.

